# In Silico Prediction of PAMPA Effective Permeability Using a Two-QSAR Approach

**DOI:** 10.3390/ijms20133170

**Published:** 2019-06-28

**Authors:** Cheng-Ting Chi, Ming-Han Lee, Ching-Feng Weng, Max K. Leong

**Affiliations:** 1 Department of Chemistry, National Dong Hwa University, Shoufeng, Hualien 97401, Taiwan; 2 Graduate Institute of Marine Biology, National Dong Hwa University, Pingtung 94450, Taiwan; 3 Department of Basic Medical Science, Center for Transitional Medicine, Xiamen Medical College, Xiamen 361023, China

**Keywords:** parallel artificial membrane permeability assay (PAMPA), in silico, two-QSAR, hierarchical support vector regression, partial least square, effective permeability coefficient (*P*_e_)

## Abstract

Oral administration is the preferred and predominant route of choice for medication. As such, drug absorption is one of critical drug metabolism and pharmacokinetics (DM/PK) parameters that should be taken into consideration in the process of drug discovery and development. The cell-free in vitro parallel artificial membrane permeability assay (PAMPA) has been adopted as the primary screening to assess the passive diffusion of compounds in the practical applications. A classical quantitative structure–activity relationship (QSAR) model and a machine learning (ML)-based QSAR model were derived using the partial least square (PLS) scheme and hierarchical support vector regression (HSVR) scheme to elucidate the underlying passive diffusion mechanism and to predict the PAMPA effective permeability, respectively, in this study. It was observed that HSVR executed better than PLS as manifested by the predictions of the samples in the training set, test set, and outlier set as well as various statistical assessments. When applied to the mock test, which was designated to mimic real challenges, HSVR also showed better predictive performance. PLS, conversely, cannot cover some mechanistically interpretable relationships between descriptors and permeability. Accordingly, the synergy of predictive HSVR and interpretable PLS models can be greatly useful in facilitating drug discovery and development by predicting passive diffusion.

## 1. Introduction

**Figure 1:**
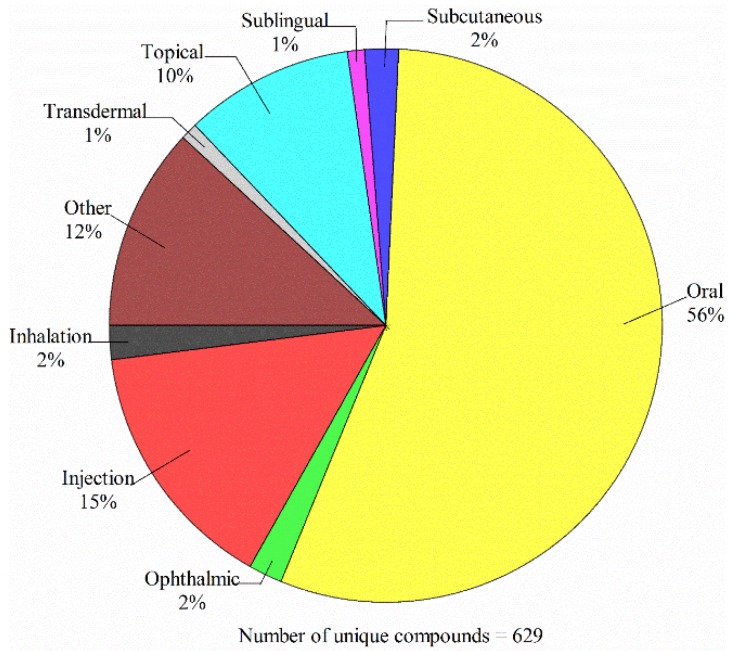
Administration routes and the corresponding ratios for those unique drugs approved by the FDA in 2018.

The oral route is the simplest and most convenient means for administrating drugs [1]. As such, oral administration is the most prevalent route of drug administration that can be manifested by Figure 1, which displays the administration routes and the corresponding ratios for 629 unique compounds approved by US FDA in 2018 based on the analysis of FDA data (https://www.accessdata.fda.gov/scripts/cder/daf/index.cfm). Accordingly, absorption is one of critical factors in absorption, distribution, metabolism and excretion, and toxicity (ADME/Tox) profiling in the process of drug discovery and development [2]. More importantly, poor absorption can make a partial contribution to clinical failures [3]. For instance, it has been observed that curcumin, which is an active ingredient extracted from the root of *Curcuma longa*, has the potential to treat Alzheimer’s disease, cancer, and diabetes as observed [4]. However, its practical applications have been severely hampered by its poor absorption [5,6].

To exert the efficacy, an orally administered drug first needs to be dissolved in the stomach fluids and then absorbed by the digestive system. Drug absorption primarily takes place in the small intestine, in which drugs can penetrate the epithelial cell layer of the small intestine in order to enter the circulatory system and thus be transported to the intended molecular target [7]. Accordingly, drug absorption is an extremely complex process that can be dominated by drug formulation and a number of physicochemical and physiological factors. The former includes solubility, stability, hydrophobicity, ionization state, and p*K*_a_, and the latter is the function of gastrointestinal (GI) pH, the gastric emptying and intestinal transit times, diameter, length, and surface area, for instance [8].

It is of necessity and great importance to evaluate drug absorption in the early stage of drug discovery to avoid failures in late-stage drug development and to reduce attrition rate [9]. In fact, a variety of in vivo, ex vivo, and in vitro assay systems have been devised to predict the small intestine absorption [10], of which cell-based assays Madin-Darby Canine Kidney (MDCK) cells and human colon adenocarcinoma derived cell line (Caco-2) and cell-free parallel artificial membrane permeability assay (PAMPA) [11] have been widely adopted to screen for permeability [12] and their good predictivity has been demonstrated [13]. The differences between the cell-based and cell-free systems are in that the former can go through carrier-mediated transport, viz. efflux or influx active transport, along with the passive route, whereas the latter can only take place through passive transcellular permeability [10], which is of pivotal importance since the majority of the marketed drugs are absorbed via passive diffusion [2]. Moreover, studies have demonstrated that cell-free permeability assay systems can be carried out in a high-throughput fashion, and are reliable, faster, and more economical than their cell- and tissue-based counterparts [14]. It has been demonstrated that PAMPA can modestly correlate with Caco-2 for some compounds [15]. The drug discovery paradigm, actually, has shifted to adopt PAMPA as the preliminary permeability screening to evaluate the passive diffusion and the cell-based assay as the secondary screening to characterize the mechanism of drug transport [16].

The PAMPA system principally is comprised of (a) a donor compartment, which includes the aqueous test compound and buffer, (b) an acceptor compartment, which contains buffer without test compound initially, and (c) an artificial membrane, which is constructed by phospholipid mixtures to separate the donor apartment from the acceptor compartment, and a porous hydrophobic filter, which is designated to support and stabilize the membrane, as illustrated by Figure 1 of Diukendjieva et al. [17]. One of the advantageous characteristics of PAMPA is its diverse applications since various membrane constructs can be adopted by PAMPA to mimic different physiological conditions [18] as compared with its cell-based counterparts. For instance, PAMPA can be used as a surrogate for the blood-brain barrier penetration (BBB-PAMPA) when porcine brain lipid extract is employed [19] or skin permeability (Skin-PAMPA) when the mixture of certramide, cholesterol, and stearic acid is used [20].

During the PAMPA transport process, solute molecules will travel from the bulk aqueous solution in the donor compartment through an unstirred water layer (UWL) or aqueous boundary layer (ABL), which is a liquid layer adjacent to the surface of the cell membrane [21], diffuse through the membrane, and enter into the UWL and the bulk aqueous solution in the acceptor compartment as illustrated by Figure 1 of Nielsen et al. [22]. As such, solute molecules will sequentially experience three environments, namely hydrophilic (bulk solution and UWL), hydrophobic (membrane), and hydrophilic (UWL and bulk solution) in the process of diffusion from the donor compartment into the acceptor compartment.

Furthermore, different measurements can lead to different PAMPA permeability coefficients, namely percentage flux (%*F*) or percentage transported solute (%*T*), which gauges the portion of the test compound in the acceptor compartment. The intrinsic permeability coefficient (*P*_o_), which is determined by the largest membrane permeability coefficient of an ionizable compound in its uncharged form of the compound that could be obtained. UWL permeability (*P*_U_ or *P*_UWL_), which corresponds to the maximum permeability coefficient of a compound in both compartments in presence of the UWL. The membrane permeability coefficient (*P*_m_) is the membrane permeability coefficient of the solute for D→A or A→D flux, and the apparent permeability coefficient (*P*_a_ or *P*_app_) is measured by the ratio between the flux and solute concentration in the donor compartment. The effective permeability coefficient (*P*_e_) is essentially identical to *P*_a_ when the amount of solute trapped in the membrane and/or A→D flux is not corrected or *P*_m_ when the system is infinitely stirred with zero UWL thickness [23,24].

In comparison to in vitro and in vivo assays, *in silico* technologies can substantially facilitate drug discovery and development due to their robustness, throughput, and cost-efficiency [25]. Their most unique and advantageous characteristics are their ability to apply to virtual compounds, which are not yet synthesized [26]. As such, in silico approaches play an increasing role in ADME/Tox profiling [27]. In fact, numerous qualitative structure–activity relationship (QSAR) models have been developed to predict PAMPA permeability [7,23,28,29,30,31,32,33,34,35,36,37,38,39,40,41,42,43,44,45,46,47].

However, PAMPA permeability depends on a number of factors such as assay pH, stirring, filter porosity, UWL thickness, buffer solution, co-solvent, and system temperature in addition to the content of membrane [22,48,49,50,51,52,53]. Those factors contribute to the inhomogeneity in assay data unless the exact protocols are carried out [7], creating paramount hurdles to develop a good quantitative in silico model using the data collected from the public domain since a sound predictive model can only be built when data with the best integrity are used [54].

Most of proposed predictive models were developed by linear regression schemes such as linear partial least square (PLS) or multiple linear regression (MLR) that can explain the linear relationship between selected descriptor and biological activity [54]. However, the bilinear relationship between logarithm of the *n*-octanol–water distribution coefficient at pH 7.4 (log *D*) and PAMPA permeability was observed by Kansy et al. [55], suggesting that linear models cannot properly interpret the complex nonlinearity. Machine learning (ML) schemes, conversely, are designated to resolve the nonlinearity between input and output as manifested by the fact that ML-based models normally execute better than their linear counterparts [56]. The relationship between input and output, conversely, is difficult to be elucidated by ML approaches since they are usually regarded as a “black box” [54]. The conflict between interpretability and predictivity can be resolved by the two-QSAR approach [57], in which the predictive ML-based model is developed by the hierarchical support vector regression (HSVR) [58] scheme and the interpretable linear model is built by PLS. Herein, the objective of this study was to predict the PAMPA effective permeability to facilitate drug discovery by using the two-QSAR scheme.

## 2. Results

### 2.1. Data Partition

**Figure 2:**
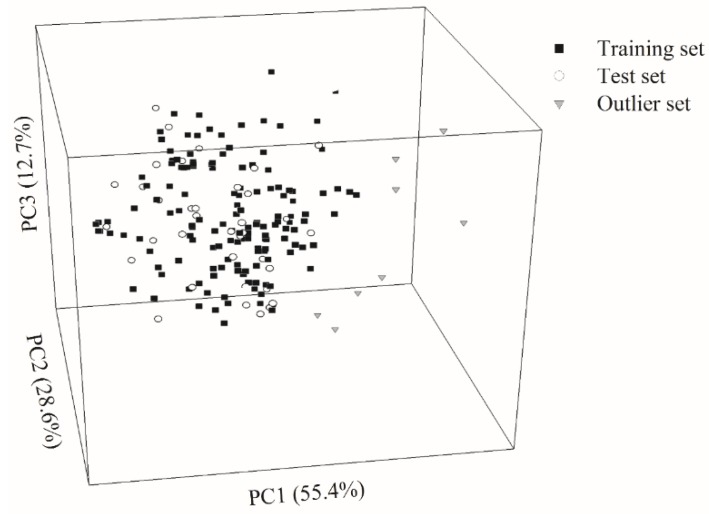
Molecule distribution for the molecules selected for this study in the training set (solid square), test set (open circle), and outlier set (grey triangle) in the chemical space spanned by three principal components.

The Kennard–Stone (KS) algorithm was adopted to assign 146 and 36 molecules into the training set and test set, respectively, with a ca. 4:1 ratio. Figure 2 displays the projection of all molecules included in this study in chemical space, spanned by the first three principal components (PCs), which rendered 96.7% of the variance in the original data. It can be observed that both data sets exhibited high degrees of similarity in the chemical space. Furthermore, the high levels of biological and chemical similarity between both sets can also be observed from Figure S1, which displays the histograms of log *P*_e_, molecular weight (MW), log *P*, log *D*, polar surface area (PSA), fractional polar surface area (FPSA), and dipole moment (*μ*) in density form for the training and test samples. Thus, it can be asserted that there was no substantial bias in data partition.

Those designated outliers are completely placed outside the perimeter of the training set in the chemical space as illustrated in Figure 2, suggesting that they were very dissimilar from those training samples [59]. Additionally, the log *P* and log *D* distribution patterns (Figure S1) also confirm their dissimilarity. In fact, the distinctions between outliers and the others can be manifested by the fact that the outliers contained more than 26 carbon atoms and more than 34 hydrogen atoms as compared with the other molecules. As such, those outliers are distant from the model applicability domain (AD) and they can be used as a good means to gauge the robustness of a predictive model.

### 2.2. HSVR

Of all generated SVR models based on a variety of descriptor combinations and runtime parameters, two SVR models, symbolized by SVR A and SVR B, were compiled to construct the SVR ensemble, which, in turn, was subjected to regression by another SVR to produce the HSVR model. The optimal runtime parameters of SVR A, SVR B, and HSVR, are listed in Table S1.

**Figure 3:**
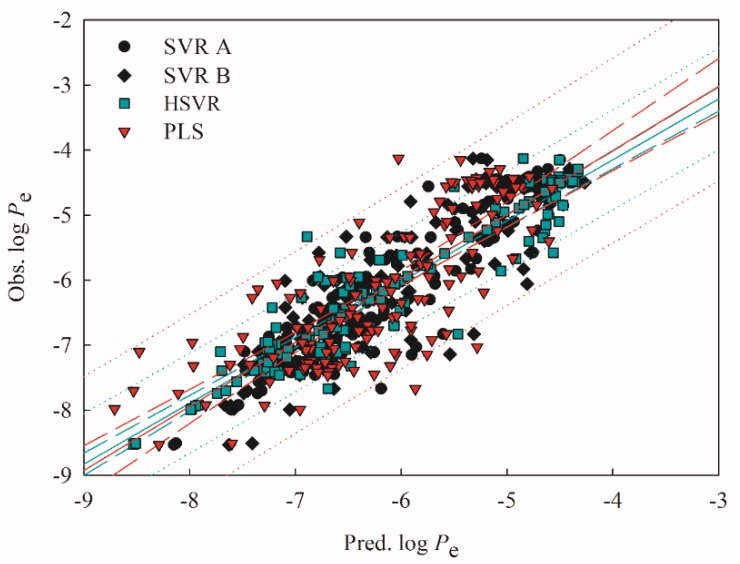
Observed log of the effective permeability coefficient (*P*_e_) vs. the log *P*_e_ predicted by SVR A (solid circle), SVR B (solid diamond), hierarchical support vector regression (HSVR; green square), and partial least square (PLS; red triangle) for the molecules in the training set. The green and red solid lines, dashed lines, and dotted lines correspond to the HSVR and PLS regressions of the data, 95% confidence intervals for the HSVR and PLS regressions, and 95% confidence intervals for the prediction, respectively.

**Figure 4:**
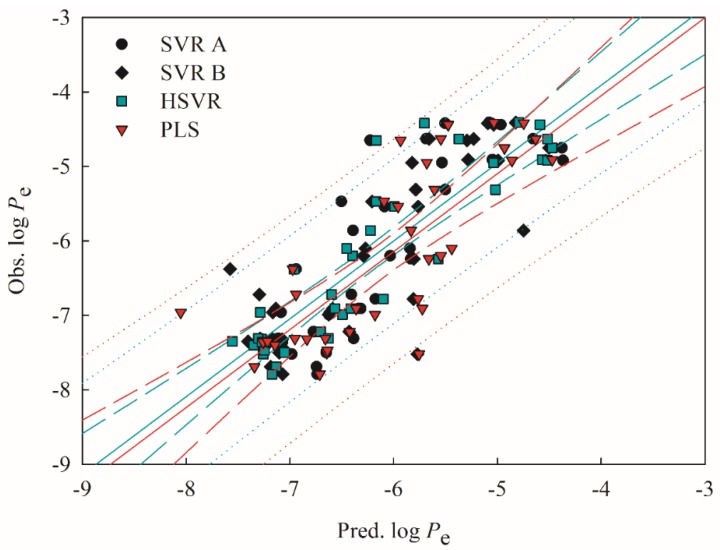
Observed log *P*_e_ vs. the log *P*_e_ predicted by SVR A (solid circle), SVR B (solid diamond), HSVR (green square), and PLS (red triangle) for the molecules in the test set. The green and red solid lines, dashed lines, and dotted lines correspond to the HSVR and PLS regressions of the data, 95% confidence intervals for the HSVR and PLS regressions, and 95% confidence intervals for the prediction, respectively.

Both SVR A and SVR B adopted different combinations of descriptors (Table 1), suggesting that they are local models per se as compared with HSVR, which is a global model per se. Accordingly, HSVR generally generated the medium deviations as compared with its counterparts in the ensemble (Table S2). Furthermore, it can be found from Figure 3 and Figure 4, which display the scatter plots of observed vs. predicted log *P*_e_ values in the training set and test set, respectively, that the distances between the predictions by HSVR and regression line were between those yielded by both SVR models in general.

HSVR, nevertheless, produced the smallest residuals in some cases. The predictions of acyclovir (compound **21**) by SVR A, SVR B, and HSVR, for instance, gave rise to absolute residuals of 0.38, 0.25, and 0.00, respectively. Statistically, HSVR executed better than both SVR models in the ensemble in the training set and test set as indicated by those parameters listed in Table 2 and Table 3. For instance, HSVR yielded the largest correlation coefficient *r*^2^, 10-fold cross-validation correlation coefficient ($q_{\mathrm{CV}}^{2}$), and *q*^2^ (0.88, 0.80, and 0.79) the smallest differences between *r*^2^ and $q_{\mathrm{CV}}^{2}$ (0.08) and between *r*^2^ and *q*^2^ (0.09), and the smallest maximum residual (Δ_Max_) and root mean square error (RMSE) in both data sets, suggesting that HSVR is highly predictive and well trained. In addition, SVR A, SVR B, and HSVR yielded the $\langle r_{s}^{2}\rangle$ values of 0.06, 0.06, and 0.03, respectively (Table 2) when subjected to the *Y*-scrambling test. Thus, it can be asserted that there is little chance correlation in those SVR models because of their nearly zero values of $\langle r_{s}^{2}\rangle$ [60].

**Figure 5:**
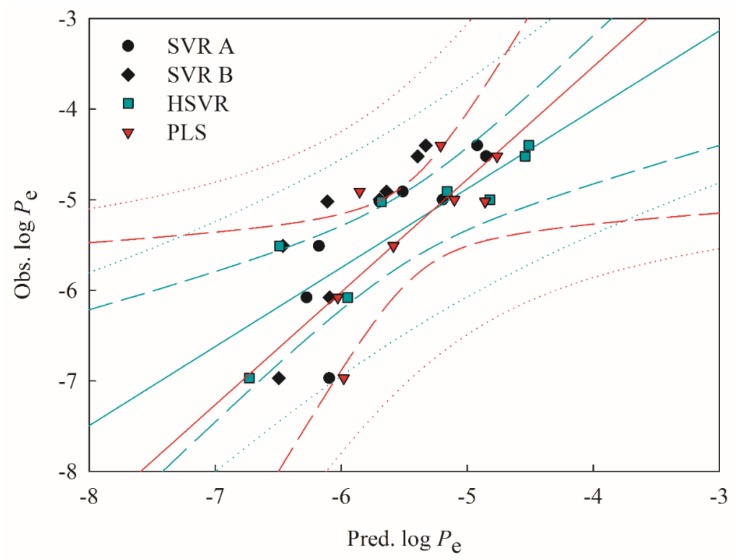
Observed log *P*_e_ vs. the log *P*_e_ predicted by SVR A (solid circle), SVR B (solid diamond), HSVR (green square), and PLS (red triangle) for the molecules in the outlier set. The green and red solid lines, dashed lines, and dotted lines correspond to the HSVR and PLS regressions of the data, 95% confidence intervals for the HSVR and PLS regressions, and 95% confidence intervals for the prediction, respectively.

Table 4 lists the static parameters evaluated by the derived models and Figure 5 displays the scatter plots of observed vs. predicted log *P*_e_ values in the outlier set. HSVR even showed more noticeable predominance as indicated by those statistical evaluations when applied to the molecules in the outlier set. For instance, SVR A, SVR B, and HSVR yielded the RMSE values of 0.56, 0.79, and 0.44, respectively. The better performance of HSVR in the outlier set can be plausibly attributed to the fact that HSVR has a broad applicability domain as compared with its counterparts in the ensemble and HSVR is more robust, which is of crucial significance to a predictive model [61].

### 2.3. PLS

The linear PLS model (Equation (1)) was constructed by collectively combining those descriptors adopted by the SVR models in the SVR ensemble (Table 1). Table S2 lists the prediction results of the molecules in the training set, test set, and outlier set, and Table 2, Table 3 and Table 4 summarize the corresponding statistical evaluations, respectively.

(1) $$\begin{array}{lll} \log P_{e} & = & 0.238253\times \log P+0.228889\times \log D-0.215243\times \mathrm{PSA}\\ & & -0.24652\times \mathrm{FPSA}-0.157312\times \mu -6.13473 \end{array}.$$

The PLS model gave rise to an *r*^2^ value of 0.61, which is lower than those produced by SVR A, SVR B, and HSVR, denoting its mediocre performance in the training set. It can be observed from Figure 3 that most of the points predicted by PLS generally had the largest distances from the regression line as compared with SVR A, SVR B, and HSVR, consequently it produced the largest Δ_Max_ (1.90), mean absolute error (MAE; 0.58), standard deviation (*s*; 0.38), and RMSE (0.70) (Table 2). However, PLS generated a $q_{\mathrm{CV}}^{2}$ of 0.76, which is not only larger than its *r*^2^ but much better than the ones produced by SVR A and SVE B, suggesting that PLS was well-trained as compared with both SVR models in the ensemble. Similar to its SVR counterparts, PLS also presents no result of chance correlation as manifested by its $\langle r_{s}^{2}\rangle$ (0.06).

PLS was the worst predictive model in the test set as manifested by its largest Δ_Max_ (1.77), MAE (0.61), *s* (1.40), and RMSE (0.73) (Table 3). However, PLS yielded a *q*^2^ value of 0.61, which was the same as its *r*^2^ counterpart in the training set, also suggesting that PLS was well-trained since it would have otherwise produced a substantial difference. In addition, the fact that PLS showed similar performance in the training set and test set indicates that there was no bias in data partition chemically and biologically since it would otherwise have given rise to substantial performance difference.

When applied to the outliers, PLS produced a *q*^2^ value of 0.54, which was smaller than those calculated by the SVR models (Table 4). However, it is of interest to note that PLS gave rise to a Δ_Max_ of 0.99, which was larger than the one produced by SVR A (0.87) and smaller than the one calculated by SVR B (1.09). Other statistical parameters also suggest that PLS executed better than either one of SVR models in the ensemble and worse than the other. However, HSVR still functioned better than PLS in every aspect.

### 2.4. Predictive Evaluations

**Figure 6:**
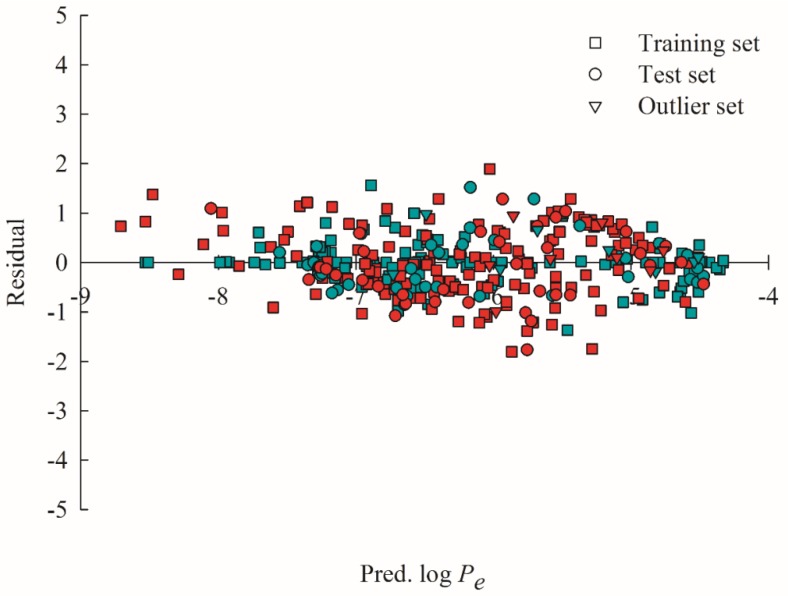
Residual vs. the log *P*_e_ predicted by HSVR (green) and PLS (red) in the training set (square), test set (circle), and outlier set (triangle).

It can be discovered from Figure 6, which displays the scatter plots of the residuals vs. the log *P*_e_ values predicted by HSVR and PLS for all molecules (i.e., training samples, test samples, and outliers) that the residuals produced by both models were approximately evenly dispersed on both sides of *x*-axis. As such, both HSVR and PLS unanimously gave rise to the average errors of 0.00 (Table S2), suggesting that little systematic errors were associated with both models. However, PLS generally yielded larger absolute residuals than HSVR as manifested by their mean absolute errors (0.28 vs. 0.58).

The derived HSVR and PLS models were further evaluated by combining the most stringent validation requirements collectively suggested by Golbraikh et al. [62], Ojha et al. [63], Roy et al. [64], and Chirico and Gramatica [65] in the training set, test set, and outlier set (Equations (15)–(21). The results are tabulated in Table 5, from which it can be found that HSVR not only generated large statistical assessments but also fulfilled all validation requirements as compared with PLS, which only met the requirements of Equations (17) and (18) in three datasets. PLS even gave rise to a negative ${r^{\prime }}_{o}^{2}$ (–0.46) in the outlier set. Accordingly, it can be asserted that HSVR outperformed PLS in every statistical aspect.

### 2.5. Mock Test

**Figure 7:**
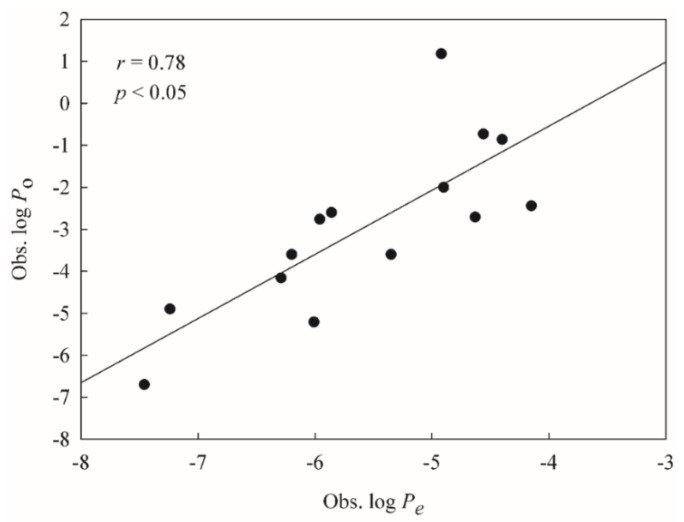
The observed log of the intrinsic permeability coefficient (*P*_o_) values vs. the observed log *P*_e_ values.

To mimic real world challenges, the developed HSVR and PLS models were further tested by a number of drugs measured by Huque et al. [47], of which 14 were also adopted in this study, providing a good way to calibrate the challenging system. Nevertheless, Huque et al. measured the *P*_o_ values using the filter costed with 2% dioleoylphosphatidylcholine (DOPC) in contrast to the compounds selected in this study, whose *P*_e_ values were assayed using the filter coated with 1% lecithin, indicating the fact that those drugs assayed by Huque et al. are not eligible as the second external set or test set due to the fact those validation criteria (vide supra) cannot be applied to these compounds. The subsequent correlation between both measured systems (i.e., log *P*_o_ vs. log *P*_e_) was initially established and inspected based on those common 14 molecules and the resulted scattered plot is displayed in Figure 7. It can be observed that both systems were modestly correlated with each other well with an *r* value of 0.78, suggesting that it is plausible to challenge the derived HSVR and PLS models with those novel compounds assayed by Huque et al.

**Figure 8:**
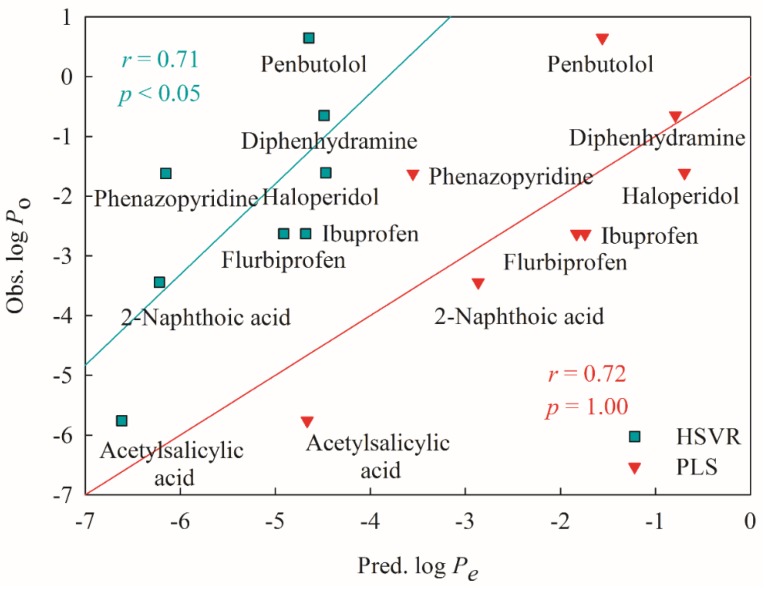
The observed log *P*_o_ values vs. the log *P*_e_ values predicted by HSVR (green square) and PLS (red triangle), and their regression lines.

Figure 8 illustrates the tested results of eight novel drugs. It can be observed that the *r* values between experimental log *P*_o_ and predicted log *P*_e_ were 0.71 and 0.72 obtained from HSVR and PLS, respectively, seemingly suggesting that both HSVR and PLS can almost reproduce the experimental observations. Nevertheless, the slope produced in the calibration system was 1.53 (Figure 7), whereas HSVR and PLS produced the slopes of 1.52 and 1.00, respectively, in the mock test. More importantly, the *p* values produced by HSVR and PLS were <0.05 and 1.00, respectively. Thus, it can be asserted that HSVR performed better than PLS in the mock test.

## 3. Discussion

PAMPA permeability takes place through a series of processes when solute molecules travel from the donor compartment into the acceptor compartment, which are governed by a number of factors such as solute–solute, solute–solvent, and solute–membrane interactions. Physico-chemically, the environment inside the membrane is non-polar and hydrophobic, whereas that outside the membrane is polar and hydrophilic per se [66]. Accordingly, hydrophobicity, which can be represented by log *P* and log *D* [67], play a significant role in PAMPA permeability that can be manifested by the fact that most of published models have adopted either descriptors.

In fact, both log *P* and log *D* were also included in this study (Table 1) and PLS derived in this investigation also gave positive coefficients to both descriptors (Equation (1)) suggesting that PAMPA permeability increases with both descriptors that is consistent with most of published models. Nevertheless, it can be argued that both HSVR and PLS can be possibly yielded by chance correlation since both descriptors can represent hydrophobicity. The correlation coefficient (*r*) between log *P* and log *D* was merely 0.66 for all compounds selected in this study, suggesting that the probability of spurious correlations was actually small [68]. More importantly, there is some subtle difference between log *P* and log *D* since the former reflects only the intrinsic hydrophilicity of neutral molecules whereas the latter takes into account not only the ionization effect of ionizable compounds but the actual hydrophilicity [69]. This indicates that it is of necessity to adopt both hydrophobic descriptors to render hydrophobicity for different scenarios when there are diverse samples in the collection.

It has been observed by Verma et al. that PAMPA permeability initially increases with the increase of log *P* to a certain value and then decreases afterwards [32]. Moreover, Akamatsu et al. observed that log *P* can be positively or negatively correlated with PAMPA permeability when permeability values are relatively low and high, respectively [23]. Similar nonlinear relationship between log *D* and PAMPA permeability has also been observed as shown by Figure 7 of Kansy et al. [55]. The discrepancy between linear and non-linear relationships can be realized by the fact that the more hydrophobic solutes are, the easier solutes can approach to the hydrophobic membrane when the solutes are entering the membrane zone, leading to a positively linear relationship. However, the more hydrophobic solutes will experience stronger interactions between solutes and the membrane as well as stronger repelling forces exerted by solvent molecules in the acceptor compartment when the solutes escape the membrane, producing an inverse relationship between hydrophobicity and permeability. Thus, too hydrophilic compounds cannot easily cross the cell membrane because of hydrophobic nature of membrane, whereas too hydrophobic ones can easily stay trapped in the cell membrane [16]. Accordingly, hydrophobicity plays a perplexing role in passive diffusion. More complexity can be introduced when taking into account the hydrophobicity of functional groups within a solute molecule since it has been suggested that solutes with both hydrophobic and hydrophilic moieties will go through a more complicated pathway than those that do not have [70].

The descriptors PSA and *μ* were adopted by SVR B and SVR A, respectively (Table 1) and given by negative coefficients by PLS (Equation (1)), suggesting that both PSA and *μ* can reduce the passive diffusion. Such observations are actually consistent with the results obtained by Iyer et al. [71] qualitatively. The selection of both descriptors can be justified by the fact that both descriptors represent the molecular polarity [72]. A greater PSA, dipole, and polarity can produce stronger interactions between solute and solute molecules and between solute and solvent molecules. As such, it will require more desolvation energy when the more polar solutes enter the lipophilic phase of membrane from the donor compartment, making them energetically less favorable and less permeable consequently. Conversely, larger solvation energy will be released once the more polar solutes escape the membrane and re-enter the bulk solution in the donor compartment. Accordingly, the nonlinear relationship between polarity and passive diffusion can be expected and the performance of the linear PLS model was worse than that of the ML-based HSVR model. It should be also noted that both PSA and *μ* were enumerated by the more sophisticated density functional theory (DFT) method and atomic charge calculation algorithm in addition to the consideration of solvent effects in this study. Those factors could profoundly affect both descriptors.

Thus, it can be asserted that those descriptors mentioned above were designated to render various parts of the complex passive diffusion—from the initial desolvation in the donor compartment to the final solvation in the acceptor compartment. The corresponding coefficients given by the PLS model can actually reflect the contributions of those selected descriptors prior to the entrance of solute into the membrane. Their contributions go into the opposite direction once solute buds from membrane into the acceptor compartment, viz. attraction becoming repelling and vice versa, producing enormous prediction errors by PLS consequently. HSVR, conversely, can properly describe such a complex process. As such, HSVR performed better than PLS in every aspect.

It is seemingly unusual to note that the descriptor FPSA was selected by SVR A and yet has hitherto not been adopted by any published model. However, FPSA was modestly correlated with PSA with an *r* value of 0.79 for all molecules included in this study. As such, it is plausible to replace PSA by FPSA that is consistent with the observation, in which the replacement did not result in substantial change in model performance [73]. It can be argued that the probability of spurious correlations can be increased by the inclusion of FPSA due to the modest association between FPSA and PSA [68] that, actually, is not applicable in this study since FPSA and PSA were separately adopted by SVR A and SVR B. In another word, neither of descriptors was simultaneously employed by the same SVR model. More importantly, the empirical observation has indicated that SVR A and SVR B with the selections of FPSA and PSA, respectively unanimously showed better performance than those with the selections of PSA only, FPSA only, as well as FPSA by SVR B and PSA by SVR A (data not shown). This presumably was due to the fact that the selected descriptors were not completely orthogonal to one another, viz. not completely independent, leading to the descriptor–descriptor interaction as we defined, in which the synergy among some descriptors can improve the model performance, especially for the ML-based nonlinear models.

**Figure 9:**
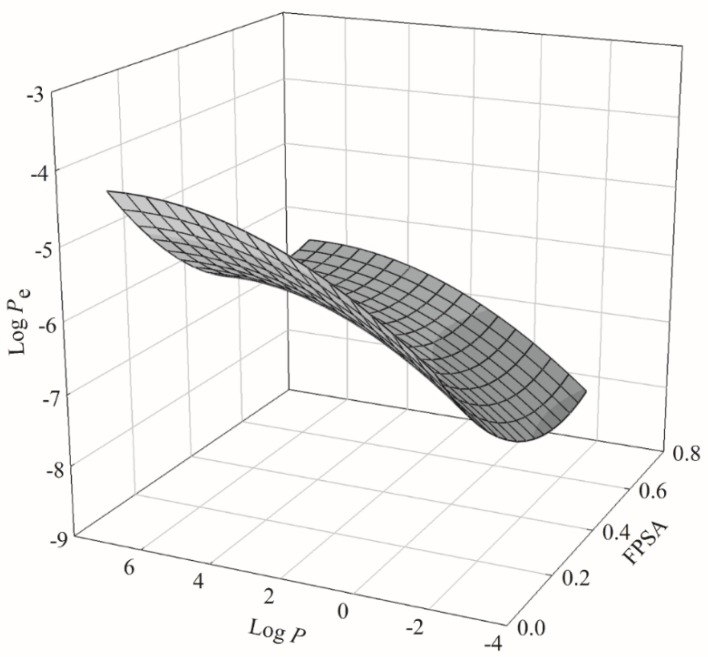
Relationship among log *P*_e_, fractional polar surface area (FPSA), and log *P* in 3D presentation.

The PLS model gave a negative coefficient to FPSA (Table 1), which was similar to the PSA coefficient. As such, it is plausible to expect FPSA played a similar role in PAMPA permeability as PSA did. This is illustrated by Figure 9, which displays the 3D plot of log *P*_e_, FPSA, and log *P*. It can be found that log *P*_e_ initially decreased with increased FPSA to a certain value and then increased afterwards, whereas log *P* behaved otherwise. In addition, the nonlinear relationship between log *P*_e_ and log *P* as mentioned above can also be observed.

Various criteria have been proposed to distinguish highly permeable compounds from poorly permeable ones. For instance, it has been observed by Kelder et al. that administrate drugs with PSA > 120 Å^2^ were poorly absorbed, whereas those with PSA < 60 Å^2^ were well absorbed [74]. Hou et al. have suggested a looser threshold (PSA > 140 Å^2^) to identify the poorly permeable compounds. Compounds collected in this study were classified as having high and low permeability if their log *P*_e_ values were ≥−6.0 and <−6.0, respectively, as suggested by Diukendjieva et al. [44] to verify the observation made by Kelder et al. The analysis of collected compounds indicated that 100% of compounds were poorly permeable and 75% of compounds were well permeable when their PSA values were >120 Å^2^ and <60 Å^2^, respectively, which is completely consistent with the observation made by Kelder et al. Only 92% of compounds were poorly permeable when the threshold of PSA was set to >110 Å^2^, suggesting that the threshold PSA > 120 Å^2^ is efficiently enough to characterize the poorly permeable compounds.

In addition, Zhu et al. have postulated that compounds with 0.0 < log *P* < 5.0 or −0.5 < log *D* < 4.5 are most likely to be absorbed [75]. Only 52% and 56% of compounds selected in this investigation showed to be well permeable, respectively, when applied to both criteria. The accuracy was dropped to 50% when both criteria were combined. Nevertheless, the accuracy was increased to 74% when the thresholds were set to be PSA < 60 Å^2^ and 0.0 < log *P* < 5.0, 81% when PSA < 60 Å^2^ and −0.5 < log *D* < 4.5, and 81% when PSA < 60 Å^2^, 0.0 < log *P* < 5.0, and −0.5 < log *D* < 4.5, after combining those criteria proposed by Zhu et al. and Kelder et al. It can be observed that the combination of PSA and log *D* factors can identify more permeable compounds than either one of them. Nevertheless, the log *P* factor makes no difference that can be plausibly attributed to the different natures of log *P* and log *D* in rendering hydrophobicity (vide supra). The selection of PSA and log *D* as the identification characteristics is actually consistent with the observation made by Flaten et al. [76].

**Figure 10:**
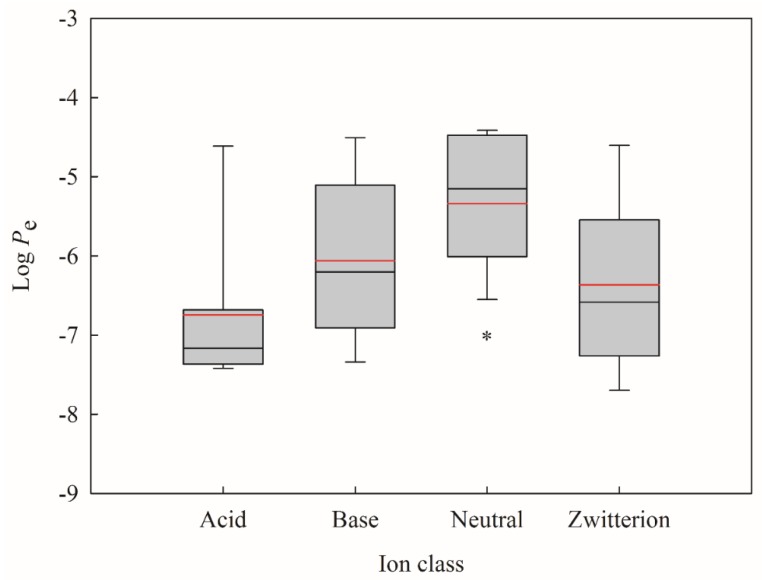
Box plot of log *P*_e_ values for different ion classes, where the boxes represent the distribution of log *P*_e_ from the 25th to the 75th percentile, the black and red lines depict the median and mean values, the whiskers denote the minimum and maximum values, and the asterisk indicates significant difference between neutral and the others (*p* < 0.05).

It has been suggested that different permeability models should be developed for different ion classes [41]. Moreover, it has been identified that neutral compounds can more easily cross the hydrophobic membrane as compared with the other ion classes [2]. Thus, all molecules selected in this study were subjected to ion class analysis. It can be observed from Figure 10, which displays the box plot of the log *P*_e_ minimum, maximum, mean, median, the 25th percentile, and the 75th percentile for each ion class, that log *P*_e_ values of neutral compounds are statistically higher than the other ion classes, suggesting that neutral compounds show higher PAMPA effective permeability. When taking into account the criteria of PSA and log *D*, 85% of neutral compounds were well permeable, which is slightly higher than the analysis only based on both factors (81%). Thus, it can be concluded that neutral compounds with PSA < 60 Å^2^ and −0.5 < log *D* < 4.5 are most likely to be permeable, whereas compounds with PSA > 120 Å^2^ will have greater probability of being poorly permeable.

## 4. Materials and Methods

### 4.1. Data Compilation

Only good quality sample data can be used to construct a sound predictive model [54]. A comprehensive literature search was executed to retrieve PAMPA permeability parameters from a variety of sources to maximize the structural diversity. However, PAMPA permeability is sensitive to the assay conditions (vide supra). To warrant data consistency and to minimize the variations in assay conditions among different data sources [34,38,39,40,41,42,51,55,75,77,78,79,80], only those molecules assayed by Oja and Maran [39,41,42] were selected in this study since they generated the largest quantity of data. If there were two or more available efflux ratio data for a given compound and in close range, the average values were then taken in order to warrant better consistency. Further data were cautiously curated by inspecting molecular structures to remove those molecules without definite stereochemistry. All molecules enrolled in this investigation, SMILES strings, CAS registry numbers, their corresponding logarithm *P*_e_ values, and references to the literature are listed in Table S2.

### 4.2. Molecular Descriptors

All of the molecules included in this investigation were subjected to full geometry optimization using the density functional theory (DFT) B3LYP method with the basis set 6-31G(d,p) by the Gaussian 09 package (Gaussian, Wallingford, CT) in the *n*-dodecane solvent system using the polarizable continuum model (PCM) [81,82] to mimic the experimental conditions. These real minima on the potential energy surface of those optimized geometries were confirmed by force calculations when no imaginary frequency was obtained. Additionally, atomic charges were also calculated by the molecular electrostatic potential-based method of Merz and Kollman [83] and the highest occupied molecular orbital energy (*E*_HOMO_), lowest unoccupied molecular orbital energy (*E*_LUMO_), dipole (*μ*), and absolute maximum component dipole moment (*μ*_max_) were also retrieved from the optimization calculations.

The Discovery Studio package (BIOVIA, San Diego, CA, USA) and E-Dragon (available at the web site http://www.vcclab.org/lab/edragon/) were also utilized to calculate more than 200 1D-, 2D-, 3D-molecular descriptors of those optimized molecules. These descriptors can be classified as electronic descriptors, spatial descriptors, structural descriptors, thermodynamic descriptors, topological descriptors, and E-state indices. The logarithm of the *n*-octanol–water distribution coefficient at pH 7.4, viz. log *D*, and p*K*_a_ were computed by Chemicalize (available at the Web site https://chemicalize.com/).

Furthermore, the cross-sectional area (CSA) was also calculated using the method modified by Muehlbacher et al. [84] because of its implication in membrane permeability [85]. Molecules were further categorized into four classes, namely zwitterion, base, acid, and neutral by their p*K*_a_ values. Specifically, zwitterions are those whose largest p*K*_a_ values are larger than 7 and the smallest p*K*_a_ are smaller than 7. The largest and smallest p*K*_a_ values of acids and bases are smaller and larger than 7, respectively. Neutrals only have one p*K*_a_ value.

Data screening was initially performed by removing those descriptors missing for at least one sample or displaying little or no discrimination against all samples. Furthermore, the probability of spurious correlations was reduced by constructing the Spearman’s matrix between calculated descriptors, followed by removing those descriptors with intercorrelation values of *r*^2^ > 0.80 as postulated by Topliss and Edwards [68]. However, the tighter threshold of *r*^2^ ≧ 0.64 was set in this study to further ensure the quality of developed models.

It is normal to observe that some descriptors with broader ranges outweigh those with narrower ranges due to substantial variations in magnitudes. Nevertheless, such a problem can be exonerated when the non-descriptive descriptors, viz. real variable descriptors, are normalized by centering and scaling into a more consistent range:

(2) $$\chi_{ij}=\left(x_{ij}-\langle x_{j}\rangle \right)/{\left[\sum_{i=1}^{n}{\left(x_{ij}-\langle x_{j}\rangle \right)}^{2}/\left(n-1\right)\right]}^{1/2},$$

where $x_{ij}$ and $\chi_{ij}$ stand for the original and normalized *j*th descriptors of the *i*th compound, respectively; $\langle x_{j}\rangle$ represents the mean value of the original *j*th descriptor; and *n* is the number of samples.

Descriptor selection plays a predominant role in determining the performance of predictive models [86]. More training samples with more diverse structures will demand more descriptors [54], whereas it is highly possible to develop an over-trained model once there are too many selected descriptors [87]. The descriptor selection was initiated by genetic function approximation (GFA) using the QSAR module of Discovery Studio because of its effectiveness and efficiency [88], followed by the recursive feature elimination (RFE) method, in which the model development was repeatedly carried out by all but one of descriptors. The descriptors were then ranked according to their contributions to the predictive performance; and the descriptor with least contribution was rejected [89].

### 4.3. Data Partition

The collected molecules were divided into the training and test sets with an approximate 4:1 ratio as suggested [90] to develop and to verify the predictive models, respectively, using the Kennard–Stone (KS) algorithm [91] implemented in MATLAB (The Mathworks, Natick, MA, USA). In addition, the data distribution was cautiously inspected to ensure the high levels of biological and chemical similarity in both data sets since it has been suggested that a sound model can be derived only based on chemically and biologically similar training samples and test samples [92].

### 4.4. Partial Least Square

Partial least square, which can process data with collinearity among descriptors, is a generalization of regression. The advantageous characteristic of PLS, accordingly, is that PLS can handle data where the number of descriptors is larger than that of observations [93]. The developed PLS model is commonly subjected to cross-validation for testing its complexity to minimize the chance correlations [94]. The PLS model development was executed by the Partial Least Square module in the Discovery Studio package.

### 4.5. Hierarchical Support Vector Regression

Support vector machine (SVM) proposed by Vapnik et al. [95] was initially designed for classification and then implemented for regression by nonlinearly mapping the input data into a higher-dimension space, in which a linear regression is performed [96]. SVM regression or SVR takes into account not only the training error but the model complexity, whereas traditional regression algorithms build predictive models by minimizing the training error. Thus, SVM shows better performance than traditional regression methods that can be attributed to its advantageous characteristics, namely dimensional independence, limited number of freedom, excellent generalization capability, global optimum, and easy implementation [97].

The novel hierarchical support vector regression (HSVR) scheme, which was originally proposed by Leong et al. was derived from SVM [58]. One of the most unique and advantageous characteristics of HSVR is its ability to simultaneously take into consideration the characteristics of a global model, viz. broader coverage of applicability domain (AD), and a local model, viz. higher level of predictivity, that are seemingly contractionary to each other [98]. More significantly, it has been demonstrated that HSVR outperformed artificial neural network (ANN), genetic algorithm (GA), and SVM [99].

The detail of HSVR has been described elsewhere [58] and the HSVR architecture can be illustrated by Figure 1 of Leong et al. [58]. Concisely, a number of SVR models were built by the LIBSVM package (software available at http://www.csie.ntu.edu.tw/~cjlin/libsvm) using various descriptor combinations and each SVR model symbolized a local model. The model development and verification were carried out using the modules svm-train and svm-predict, respectively, implemented in LIBSVM. The regression modes, namely *ε*-SVR and *γ*-SVR, were used. Of various available kernel functions, radial basis function (RBF) was adopted because of its simplicity and better performance when compared with the others [100]. The runtime parameters, namely regression modes *ε*-SVR and *ν*-SVR, the associated *ε* and *ν*, cost *C*, and the kernel width *γ*, were automatically scanned by the systemic grid search algorithm using an in-house Perl script, in which all parameters were parallelly alternated.

Initially, two SVR models were adopted to build an SVR ensemble (SVRE), which, in turn, was further subjected to regression by another SVR to yield the final HSVR model. The two-member SVREs were continuously constructed until the HSVR model performed well. The three- or even four-member ensembles, otherwise, were developed by adding one or more SVR models, respectively, if all two-member ensembles failed to execute well. The descriptor selection and ensemble assembly were primarily ruled by the principle of Occam’s razor [101] by selecting the least numbers of descriptors and SVR models.

### 4.6. Predictive Evaluation

The predictivity of a produced model was assessed by several statistic parameters. The coefficients *r*^2^ and *q*^2^ in the training set and external set, respectively, for the linear least square regression were computed by the following equation

(3) $$r^{2},q^{2}=1-\sum_{i=1}^{n}{\left({\hat{y}}_{i}-y_{i}\right)}^{2}/\sum_{i=1}^{n}{\left(y_{i}-\langle \hat{y}\rangle \right)}^{2},$$

where ${\hat{y}}_{i}$ and $y_{i}$ are the predicted and observed values, respectively; and $\langle \hat{y}\rangle$ and *n* represent the average predicted value and the number of samples in the data set, respectively.

Furthermore, the residual Δ*_i_*, which is the difference between $y_{i}$ and ${\hat{y}}_{i}$, was computed:

(4) $$\Delta_{i}=y_{i}-{\hat{y}}_{i}.$$

The root means square error (RMSE) and the mean absolute error (MAE) for *n* samples in the data set were calculated:

(5) $$\mathrm{RMSE}={\left[\sum_{i=1}^{n}\Delta_{i}^{2}/n\right]}^{1/2}$$

(6) $$\mathrm{MAE}=\frac{1}{n}\sum_{i=1}^{n}\left|\Delta_{i}\right|.$$

Furthermore, various modified versions of *r*^2^ proposed by Ojha et al. [63] were also calculated

(7) $$r_{m}^{2}=r^{2}\left(1-\sqrt{\left|r^{2}-r_{o}^{2}\right|}\right),$$

(8) $${r^{\prime }}_{m}^{2}=r^{2}\left(1-\sqrt{\left|r^{2}-{r^{\prime }}_{o}^{2}\right|}\right),$$

(9) $$\langle r_{m}^{2}\rangle =\left(r_{m}^{2}+{r^{\prime }}_{m}^{2}\right)/2,$$

(10) $$\Delta r_{m}^{2}=\left|r_{m}^{2}-{r^{\prime }}_{m}^{2}\right|,$$

where the correlation coefficient $r_{o}^{2}$ and the slope of the regression line *k* were derived from the regression line (predicted vs. observed values) through the origin, whereas ${r^{\prime }}_{o}^{2}$ was computed from the regression line (observed vs. predicted values) through the origin.

The generated model was further subjected to 10-fold cross-validation using the function provided by the programs instead of the commonly used leave-one-out because of its better performance [102], yielding the correlation coefficient of 10-fold cross validation $q_{\mathrm{CV}}^{2}$. In addition to internal cross-validation, the derived models were also internally validated by the *Y*-scrambling test [54], which was carried out by randomly permuting the log *P*_e_ values, viz. *Y* values, to refit the previously built models while the descriptors were remained unaltered, giving rise to the correlation coefficient $r_{s}^{2}$. Finally, the average correlation coefficient $\langle r_{s}^{2}\rangle$ was produced after 25 times of scrambling as proposed [60].

Furthermore, QSARINS [103,104] was used to calculate the correlation coefficients $q_{F1}^{2}$, $q_{F2}^{2}$, and $q_{F3}^{2}$ and concordance correlation coefficient (*CCC*) for the external data set [105].

(11) $$q_{F1}^{2}=1-\sum_{i=1}^{n_{\mathrm{EXT}}}{\left(y_{i}-{\hat{y}}_{i}\right)}^{2}/\sum_{i=1}^{n_{\mathrm{EXT}}}{\left(y_{i}-\langle y_{\mathrm{TR}}\rangle \right)}^{2},$$

(12) $$q_{F2}^{2}=1-\sum_{i=1}^{n_{\mathrm{EXT}}}{\left(y_{i}-{\hat{y}}_{i}\right)}^{2}/\sum_{i=1}^{n_{\mathrm{EXT}}}{\left(y_{i}-\langle y_{\mathrm{EXT}}\rangle \right)}^{2},$$

(13) $$q_{F3}^{2}=1-\left[\sum_{i=1}^{n_{\mathrm{EXT}}}{\left(y_{i}-{\hat{y}}_{i}\right)}^{2}/n_{\mathrm{EXT}}\right]/\left[\sum_{i=1}^{n_{\mathrm{TR}}}{\left(y_{i}-\langle y_{\mathrm{TR}}\rangle \right)}^{2}/n_{\mathrm{TR}}\right],$$

(14) $$CCC=\frac{2\sum_{i=1}^{n_{\mathrm{EXT}}}\left(y_{i}-\langle y_{\mathrm{EXT}}\rangle \right)\left({\hat{y}}_{i}-\langle {\hat{y}}_{\mathrm{EXT}}\rangle \right)}{\sum_{i=1}^{n_{\mathrm{EXT}}}{\left(y_{i}-\langle y_{\mathrm{EXT}}\rangle \right)}^{2}+{\left({\hat{y}}_{i}-\langle {\hat{y}}_{\mathrm{EXT}}\rangle \right)}^{2}+n_{\mathrm{EXT}}{\left(\langle y_{\mathrm{EXT}}\rangle -\langle {\hat{y}}_{\mathrm{EXT}}\rangle \right)}^{2}},$$

where *n*_TR_ and *n*_EXT_ represent the numbers of samples in the training set and external set, respectively; $\langle {\hat{y}}_{\mathrm{TR}}\rangle$ is the average predicted value in the training set; and $\langle y_{\mathrm{EXT}}\rangle$ and $\langle {\hat{y}}_{\mathrm{EXT}}\rangle$ stand for the average observed and predicted values in the external set, respectively.

More importantly, a model can be regarded as predictive if it can meet the most stringent criteria collectively suggested by Golbraikh et al. [62], Ojha et al. [63], Roy et al. [64], and Chirico and Gramatica [65].

(15) $$r^{2},q_{\mathrm{CV}}^{2},q^{2},q_{\mathrm{Fn}}^{2}\geq 0.70,$$

(16) $$\left|r^{2}-q_{\mathrm{CV}}^{2}\right|<0.10,$$

(17) $$\left(r^{2}-r_{o}^{2}\right)/r^{2}<0.10\;\mathrm{and}\;0.85\leq k\leq 1.15,$$

(18) $$\left|r_{o}^{2}-{r^{\prime }}_{o}^{2}\right|<0.30,$$

(19) $$r_{m}^{2}\geq 0.65,$$

(20) $$\langle r_{m}^{2}\rangle \geq 0.65\;\mathrm{and}\;\Delta r_{m}^{2}<0.20,$$

(21) CCC≥0.85,

where *r* in Equations (17)–(20) stand for the parameters *r* and *q* in the training set and external set, respectively; and $q_{\mathrm{Fn}}^{}$ in Equation (15) represents $q_{F1}^{}$, $q_{F2}^{}$, and $q_{F3}^{}$.

## 5. Conclusions

PAMPA is often used as a surrogate for preliminary assessment of drug absorption, which plays a critical role in drug bioavailability. The two-QSAR approach was employed in this investigation by integrating hierarchical support vector regression and partial least square to predict PAMPA effective permeability. The derived HSVR model showed excellent performance in the training set, test set, and even outlier set, whereas the PLS model modestly executed in those three data sets. The accuracy and predictivity of HSVR were confirmed by various statistical assessments and validation criteria. When mock tested by a group of molecules to mimic real challenges, the derived HSVR not only showed excellent performance but executed better than PLS. The outstanding persistent performance, generalization capacity, and robustness of HSVR can be attributed to its unique architecture that can simultaneously possess the advantageous characteristics of a local model and a global model, viz. broader applicability domain and higher predictivity, respectively. The linear PLS model, conversely, disclosed the interpretable relationships between some selected descriptors and permeability that is not possible by “black box” approaches. In addition, the characteristics associated with good and poor permeability were elaborated in detail. Thus, it can be asserted that this two-QSAR approach by using predictive HSVR and interpretable PLS in a synergistic fashion can be used to predict the PAMPA effective permeability and to render the relationships between selected descriptors and passive diffusion, respectively. This can be employed to facilitate drug discovery and development by predicting the passive diffusion of hit and lead compounds. In addition, this study has paved the way to understand the transport-mediated permeability and to establish quantitative structure–bioavailability relationship (QSBR) models in the future.

## Figures and Tables

**Table 1:** Descriptor selected as the input of support vector regression (SVR) models in the ensemble and their descriptions.

Descriptor	SVR A	SVR B	Description
log *P*		x ^†^	Logarithm of the *n*-octanol–water partition coefficient
log *D*	x	x	Logarithm of the *n*-octanol–water distribution coefficient at pH 7.4
PSA		x	Polar surface area
FPSA	x		The ratio of total partially positively charged molecular surface area to total molecular surface area
*μ*	x		Dipole moment for the molecule

^†^ Selected.

**Table 2:** Statistic evaluations, namely correlation coefficient (*r*^2^), maximum residual (Δ_Max_), mean absolute error (MAE), standard deviation (*s*), root mean square error (RMSE), and 10-fold cross-validation correlation coefficient ($q_{\mathrm{CV}}^{2}$ ) evaluated by SVR A, SVR B, HSVR, and PLS in the training set.

	SVR A	SVR B	HSVR	PLS
*r* ^2^	0.84	0.79	0.88	0.61
Δ_Max_	1.48	1.60	1.56	1.90
MAE	0.38	0.39	0.24	0.58
*s*	0.26	0.33	0.31	0.38
RMSE	0.46	0.51	0.39	0.70
$q_{\mathrm{CV}}^{2}$	0.57	0.14	0.80	0.76
$\langle r_{s}^{2}\rangle$	0.06	0.06	0.03	0.06

**Table 3:** Statistic evaluations, correlation coefficients *q*^2^, $q_{F1}^{2}$, $q_{F2}^{2}$, and $q_{F3}^{2}$, concordance correlation coefficient (*CCC*), maximal absolute residual (Δ_Max_), mean absolute error (MAE), standard deviation (*s*), and RMSE evaluated by SVR A, SVR B, HSVR, and PLS in the test set.

	SVR A	SVR B	HSVR	PLS
*q* ^2^	0.72	0.70	0.79	0.61
$q_{F1}^{2}$	0.70	0.70	0.79	0.60
$q_{F2}^{2}$	0.70	0.70	0.79	0.60
$q_{F3}^{2}$	0.68	0.68	0.86	0.58
*CCC*	0.80	0.82	0.88	0.74
∆_Max_	1.58	1.75	1.52	1.77
MAE	0.53	0.51	0.42	0.61
*s*	0.35	0.37	0.32	1.40
RMSE	0.63	0.57	0.52	0.73

**Table 4:** Statistic evaluations, correlation coefficients *q*^2^, $q_{F1}^{2}$, $q_{F2}^{2}$, and $q_{F3}^{2}$, concordance correlation coefficient (*CCC*), maximal absolute residual (Δ_Max_), mean absolute error (MAE), standard deviation (*s*), and RMSE evaluated by SVR A, SVR B, HSVR, and PLS in the outlier set.

	SVR A	SVR B	HSVR	PLS
*q* ^2^	0.68	0.69	0.76	0.54
$q_{F1}^{2}$	0.78	0.56	0.86	0.76
$q_{F2}^{2}$	0.52	0.04	0.70	0.49
$q_{F3}^{2}$	0.75	0.50	0.84	0.74
*CCC*	0.69	0.48	0.85	0.63
∆_Max_	0.87	1.09	0.98	0.99
MAE	0.51	0.72	0.32	0.42
*s*	0.25	0.34	0.33	0.41
RMSE	0.56	0.79	0.44	0.57

**Table 5:** Validation verification of HSVR and PLS based on prediction performance of the molecules in the training set, test set, and outlier set.

	Training Set		Test Set		Outlier Set	
	HSVR	PLS	HSVR	PLS	HSVR	PLS
$r_{o}^{2}$	0.88	0.61	0.79	0.61	0.76	0.51
*k*	1.00	1.02	1.00	1.02	0.96	0.98
${r^{\prime }}_{o}^{2}$	0.88	0.38	0.71	0.35	0.74	–0.46
$r_{m}^{2}$	0.83	0.23	0.76	0.61	0.69	0.45
${r^{\prime }}_{m}^{2}$	0.84	0.60	0.57	0.30	0.64	0.00
$\langle r_{m}^{2}\rangle$	0.83	0.42	0.67	0.45	0.67	0.23
$\Delta r_{m}^{2}$	0.01	0.29	0.19	0.30	0.05	0.45
Equation (15)	X		X		X	
Equation (16)	X		X		X	
Equation (17)	X	X	X	X	X	X
Equation (18)	X	X	X	X	X	X
Equation (19)	X		X		X	
Equation (20)	X		X		X	
Equation (21)	X		X		X	

## Supplementary Materials

Supplementary materials can be found at https://www.mdpi.com/1422-0067/20/13/3170/s1. Table S1. Optimal runtime parameters for the SVR models; Table S2. Selected compounds for this study, their names, SMILES strings, CAS numbers, observed log *P*_e_ values and predicted values by SVR A, SVR B, HSVR, and PLS, data partitions, and references; Figure S1. Histograms of: (A) observed log *P*_e_; (B) molecular weight (MW); (C) log *P*; (D) log *D* (distribution coefficient); (E) polar surface area (PSA); (F) fractional polar surface area (FPSA); and (G) dipole moment (*μ*) in density form for all molecules in the training set, test set, and outlier set.
