# Supplementary material for: In Silico Prediction of PAMPA Effective Permeability Using a Two-QSAR Approach
Source: Int J Mol Sci. 2019 Jun 28;20(13):3170. doi: 10.3390/ijms20133170 (PMC6651837; doi:10.3390/ijms20133170)
Supplement: Supplementary file 1 [file ijms-20-03170-s001.zip › ijms-527538 toconversion supplementary/Figure S1.docx]

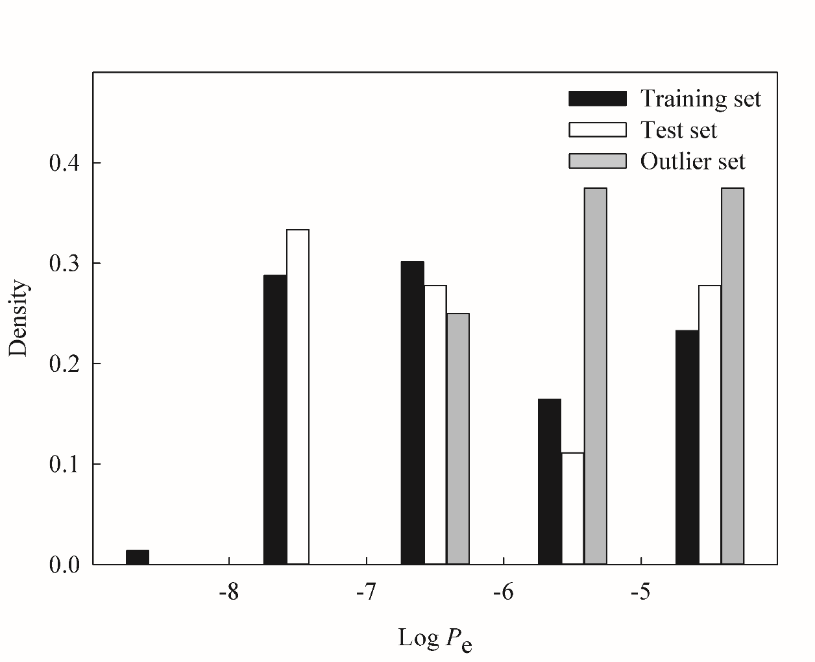


(A)


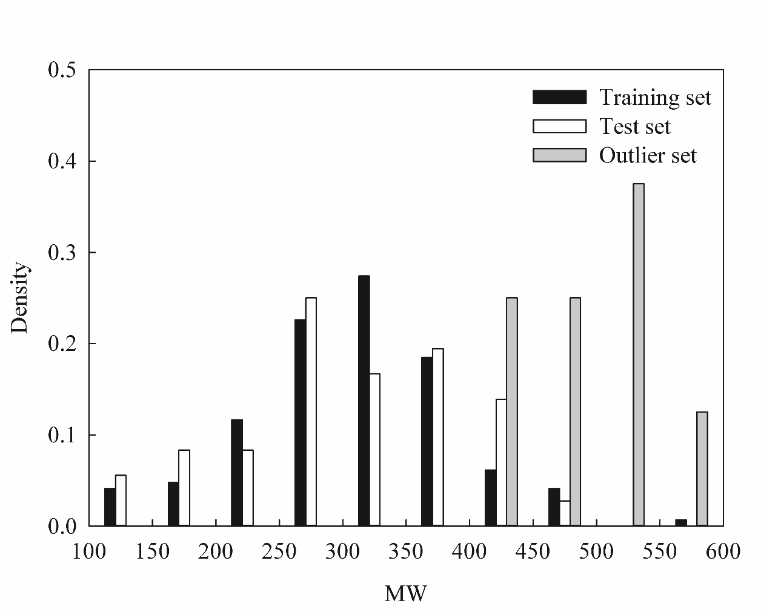


(B)


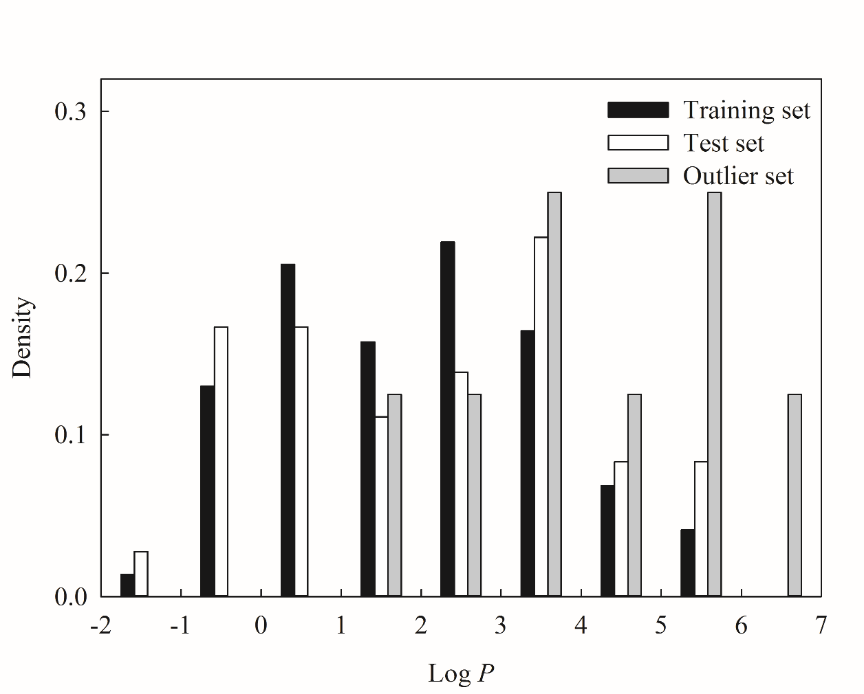


(C)


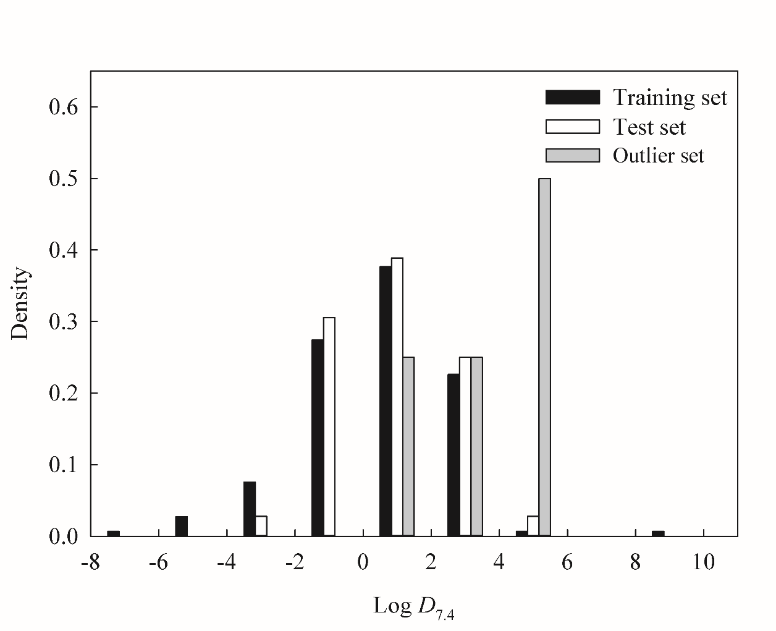


(D)


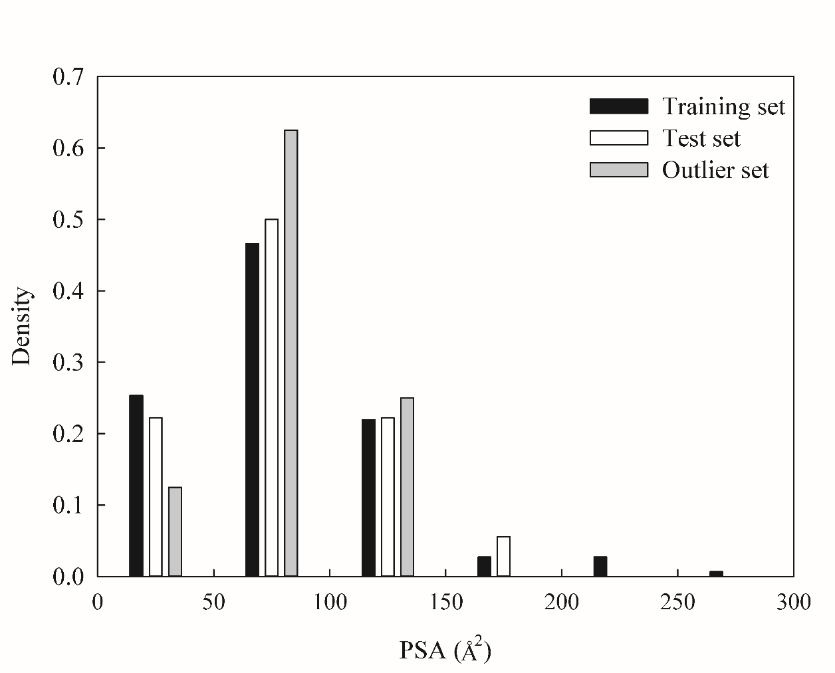


(E)


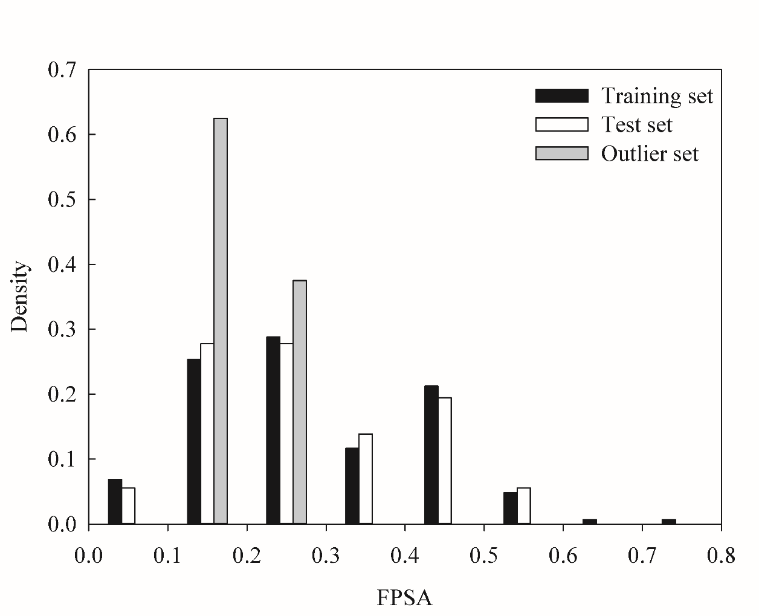


(F)


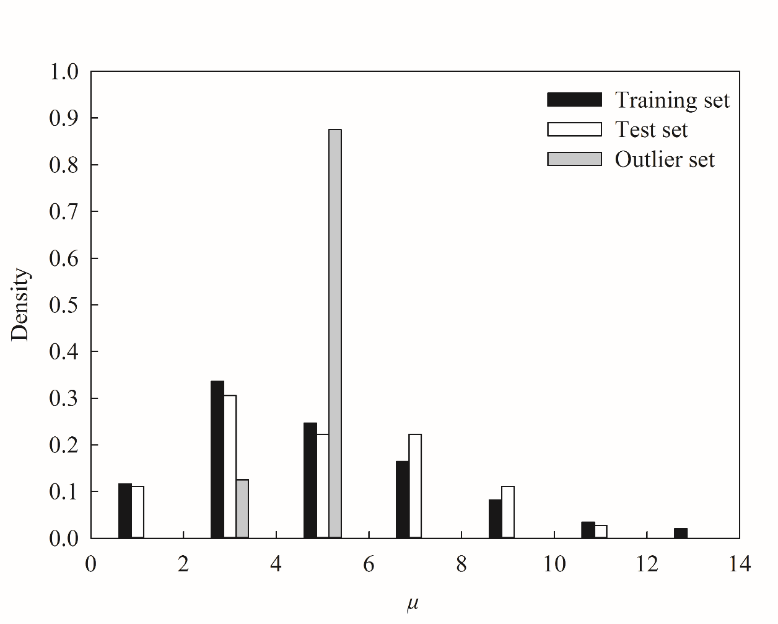


(G)

Figure 1. Histogram representation of the distributions of various descriptors for all molecules in the training set, test set, and outlier set. (A) log *P*_e_, (B) molecular weight (MW), (C) log *P*, (D) log *D*, (E) polar surface area (PSA), (F) fractional polar surface area (FPSA), and (G) dipole moment (*μ*).
